# Morphological study of the eye and adnexa in capuchin monkeys (*Sapajus* sp.)

**DOI:** 10.1371/journal.pone.0186569

**Published:** 2017-12-05

**Authors:** Danielle Nascimento Silva, Arianne Pontes Oriá, Nayone Lantyer Araujo, Emanoel Martins-Filho, Caterina Muramoto, Fernanda de Azevedo Libório, Alessandra Estrela-Lima

**Affiliations:** 1 Postgraduate Program in Animal Science in the Tropics, Federal University of Bahia, Salvador, Bahia, Brazil; 2 Department of Anatomy, Pathology and Veterinary Clinics, Federal University of Bahia, Salvador, Bahia, Brazil; 3 Researcher of the Regional Scientific and Technological Development Program of the Foundation for Research Support of the State of Bahia, Salvador, Bahia, Brazil; 4 Screening Center for Wild Animals, Brazilian Institute of the Environment and Renewable Natural Resources, Salvador, Bahia, Brazil; Wayne State University School of Medicine, UNITED STATES

## Abstract

The objective of this study was to describe the anatomic and histologic features of the *Sapajus* sp. eye, comparing similarities and differences of humans and other species of non-human primates for biomedical research purposes. Computed tomography (CT) of adnexa, eye and orbit live animal, as well as formolized pieces of the same structures of *Sapajus* sp. for anatomical and histological study were also performed. The anatomical description of the eye and adnexa was performed using the techniques of topographic dissection and exenteration. Histological fragments were fixated in buffered formalin 10%, processed by the routine paraffin inclusion technique, stained with hematoxylin-eosin and special stains. CT scan evaluation showed no differences between the live animal and the formolized head on identification of visual apparatus structures. Anatomic and histologic evaluation revealed rounded orbit, absence of the supraorbital foramen and frontal notch, little exposure of the sclera, with slight pigmentation of the exposed area and marked pigmentation at the sclerocorneal junction. Masson's Trichrome revealed the Meibomian glands, the corneal epithelium and Bowman's membrane; in the choroid, melanocytes and Bruch's membrane were observed; and in the retina, cones and rods as well as, optic nerve, the lamina cribrosa of the nerve fibers bundles. Toluidine blue highlighted the membranes: Bowman, Descemet and the endothelium; in the choroid: melanocytes; and in the retina: nuclear layers and retinal pigment epithelium. In view of the observed results *Sapajus* sp. is an important experimental model for research in the ophthalmology field, which has been shown due to the high similarity of its anatomical and histological structures with the human species.

## Introduction

The Platyrrini or neotropical primates are distributed in Central and South America [1,2] and present a wide diversity in species and staining patterns [2,3]. Due to the phylogenetic proximity to humans, the use of non-human neotropical primates as experimental models in biomedical research has been reported [4–6]. Among the species of primates, studies on the anatomy of the eye and adnexa were performed on gorilla [7], black tufted tamarin (*Callithrix penicillata*), scent monkey (*Saimiri sciureus*) and night monkey (*Aotus azarae infulatus*) [8]. The capuchin monkeys (*Sapajus* sp.) have already been used as an experimental model in comparative visual studies [9,10]. However, anatomical, histological and imaging descriptions of ophthalmic structures have not been described. Thus, the present study aims to describe the anatomical and histological features of the eye and adnexa of *Sapajus* sp. comparing the similarities and differences of the human species, for biomedical research purposes.

## Material and methods

The research protocols were approved by Ethics Commission for the use of Experimental Animals of School of Veterinary Medicine and Zootechny of the Federal University of Bahia (protocol n° 42/2015) (S1 File) and are in accordance with the Authorization and Information System on Biodiversity, Brazilian Ministry of the Environment—SISBIO file n° 51786–1 (S2 File). It was conducted in accordance with the bioethic guidelines stated by ARVO—Association for Research in Vision and Ophthalmology (National Institutes of Health Publications No. 85–23: Revised, 1985) [11].

### Animals

One live adult animal of the genus *Sapajus*, male, weighing 3 kg, from the Triage Center of Wild Animals (CETAS-IBAMA), Salvador / Bahia, was evaluated by computed tomography. The animals of the genus Sapajus, obtained from the Triage Center of Wild Animals (CETAS-IBAMA), Salvador / Bahia, were housed in outdoor enclosures (20m x 5m), divided in eight smaller enclosures, for groups of three or four capuchin monkeys. The omnivore diet consisting of assorted fruits, vegetables, meat, eggs and dog food. For environmental enrichment the enclosures are equipped with wood trunks, ropes and ladders, allowing the animals to perform a species-specific behavior and exercise the control in their environment. In addition, the objects are arranged to facilitate cleaning, capture and monitoring of the animals. The enrichment also consists in offering food wrapped in banana leaves and, mixed and frozen fruits to be offered as popsicles.

For the histological and anatomical study, three heads of capuchin monkeys (*Sapajus* sp.)–one young female, 2.1 kg (Animal 1) and two adults, one female, 2.3 kg (Animal 2) and one male, 2.6 kg (Animal 3)–that died of natural causes, without bone sense organ abnormalities, provided by CETAS. These animals were referred to the Laboratory of Veterinary Pathology of the Federal University of Bahia, where the steps of the anatomical and histological study were carried out. Therefore, 0.1 mL of 10% neutral formalin was injected and buffered at the commissures, medial and lateral conjunctiva eyelid of each eye and 0.2 mL in the posterior segment of the eye, for fixation of the internal structures. After 48 hours of fixation, macroscopic evaluation was performed.

### Procedures

#### Computed tomographic evaluation

The live animal was submitted to dissociative anesthesia protocol with ketamine (15 mg/kg) and midazolam (0.5 mg/kg), [12] followed by induction with propofol (5.0 mg/kg) and anesthetic induction was performed using isofluorane (Isofluoran^®^, Biochimico, Rio de Janeiro / RJ). The study was carried out in a cranio-caudal direction, with the animal on ventral decubitus position. Pre and post-contrast images were obtained in transverse plane (axial) with four-channel helical tomography (Asteion^™^ Super 4 edition; Toshiba^®^, São Paulo, Brazil). The test was performed with the technique parameters: rotation time of 1.0 s, voltage of 120 kV, amperage of 150 mA and cutoff thickness of 1.0 mm. The same technique was performed on the cadaver. Iodinated contrast medium (Optiray 320, manufacturer Mallinckrodt Inc^®^) was injected intravenously at the dose of 2.0 mL/kg. The images were reconstructed with the **head brain** and **bone** sharp filters by Osyrix software. The objective of this evaluation was to verify if the structures observed in the living animal were easily identified in the cadaver, to perform the macroscopic and morphometric evaluation of the visual apparatus and aid in the identification of structures macroscopically difficult to visualize.

#### Anatomical description of the adnexa and eye

The macroscopic evaluation of the visual apparatus of three cadavers was performed using a topographic dissection method and all steps were photo documented (Nikon^®^ D7000). After identification of the external structures of the eye, a sagittal skin section from the nasal region to the frontal region of the eyelids was performed to allow the skin to be folded and the exenteration technique to be used [13]. Features such as location, shape, distances and relationship between structures were described. The eye was sectioned in the dorsoventral plane, including the optic nerve, to identify the internal structures. The measurements were performed with a digital caliper rule (Mitutoyo, São Paulo, Brazil). In the living animal, a macroscopic evaluation of the visual apparatus was also performed from CT scan.

#### Anatomic description of the orbit

The anatomy of the orbit was studied in skulls of three *Sapajus* sp. The measurements were performed using a digital caliper (Mitutoyo, São Paulo, Brazil) following the adapted methodology described by Sarma [14]. Craniometric parameters of the orbital region was also considered [15]. The following orbital parameters were measured:

(I) Vertical orbital height: the perpendicular distance between the supraorbital and infraorbital margins of the orbit.
(II) Horizontal orbital width: the distance between the rostral and caudal margins of the orbital rim, that is, the distance between the point maxillofrontale (mf) and ektokonchion (ek).
(III) Orbital index = (orbital width / orbital height) x 100.
(IV) Orbital depth: distance between the optic foramen and the center of the orbital.
(V) Orbital area: 22/7 I II, where I and II are half the orbital height and width, respectively.
(VI) Bi orbital distance: point located at the outer edge of the orbit and as far away as possible from the maxillofrontale point (bi ektokonchion).
(VII) Interorbital distance:
  a) Rostral: distance between the junction of the frontolacrimal sutures at the rostral margins of both orbits (bi maxillofrontale distance).
  (b) Mid-level: distance between the supraorbital borders of both orbits (bi lacrimale—la).
  (c) Caudal: distance between the junction of the zygomatic bones at the caudal margins of both orbits (bi dakryon–d distance).

#### Processing and histological analysis of the adnexa and eye

For the histological analysis, all the structures identified in anatomical study were processed by the routine histological paraffin inclusion technique [16]. Hematoxylin-eosin (HE) staining and special staining were performed with 4μm sections: Masson's trichrome, Toluidine blue and ferric hematoxylin. At the end of this stage, the histological sections were analyzed under optical microscope, with a coupled camera, to capture the images (Zeiss Microscope, Axion Cam Scope A.1 / AX10 and Axion Cam ICc5).

## Results

### Computed tomography

#### Lacrimal gland

**Fig 1:**
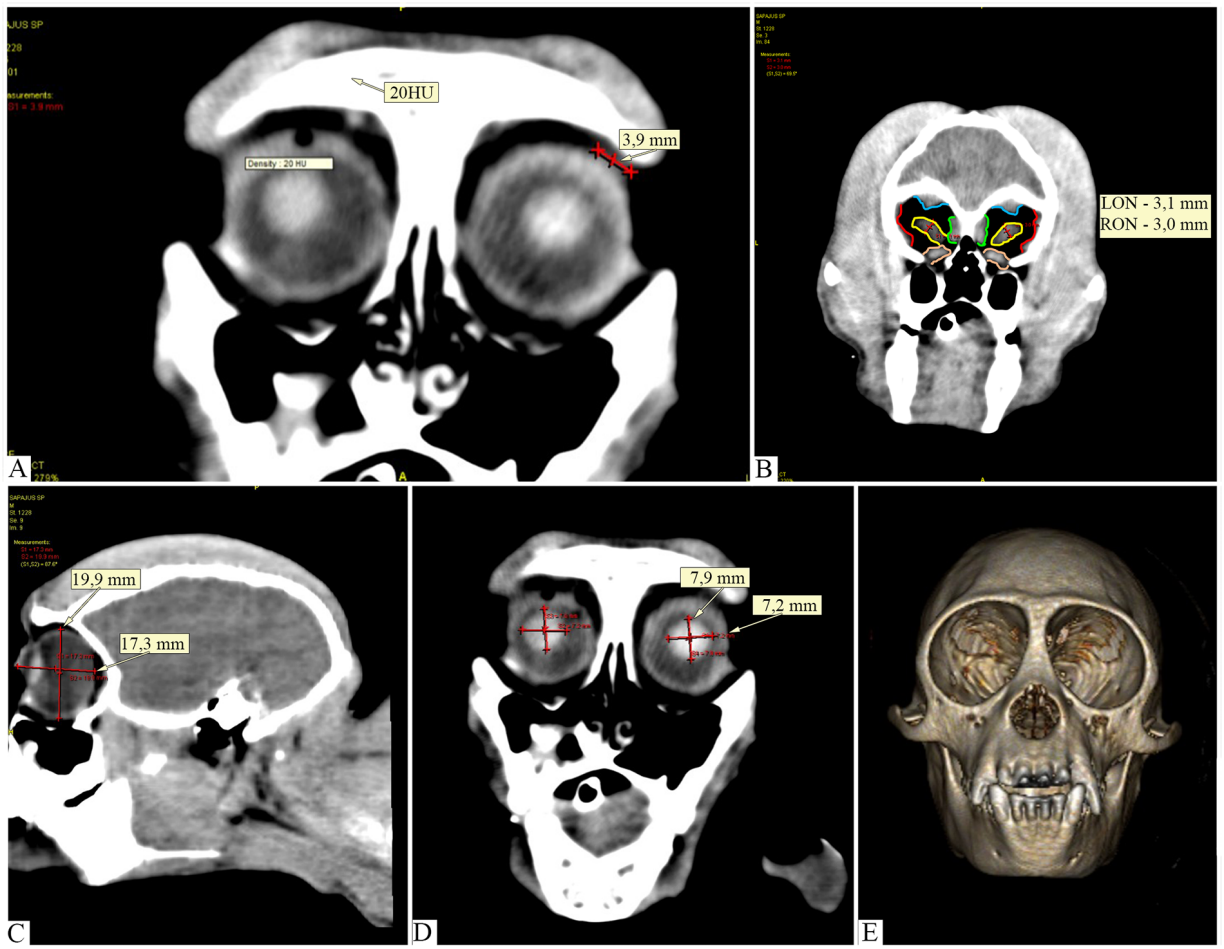
Images of computed tomography of the eye, adnexa and orbit of capuchin monkey in transverse and sagittal planes. (A) Structure with soft tissues attenuation in lacrimal gland topography. (B) Disposition of the extrinsic rectus muscles in the orbit and thickness of the optic nerves, LON: 3.1 mm and RON: 3.0 mm. (C) Sagittal plane cut in which measures of the eye are identified in the facial-caudal (17.3 mm) and dorsoventral (19.9 mm). (D) Cutting in transverse plane with lens measurement, in transverse and dorsoventral axis. (E) Image of computed tomography in three-dimensional reconstruction for bone (with suppression of soft parts) showing the closed orbit.

Identified as a small structure of undefined shape, with soft tissue density (0 to 26 HU), located in the lateral and dorsal portion of the eye, between the surface of the eye and the orbital face of the frontal bone. Dimensions: 4.0 mm wide (medial-posterior direction) and 2.0 mm thick (dorsal-ventral direction) (Fig 1A).

#### Extraocular muscles

Table 1 shows the dimensions and attenuation of the extrinsic muscles. The dorsal surface of the eye, site of insertion of the superior rectus muscles and elevator of the upper eyelid, presents as a thickening with soft tissue attenuation. A triangular structure was observed with the largest base facing the orbit surface measuring 5.0 mm x 3.0 mm and its apex directed to the eye in topography suggestive of the trochlea. The superior oblique muscle present as a thin structure and in the caudal direction of the eye, three muscles—upper rectum, upper eyelid lifter and superior oblique—unite and form a muscular structure with a triangular aspect, juxtaposed to the upper part of the orbit with attenuation of up to 60 HU.

**Table 1:** Dimensions and attenuation degrees of the adnexa and eye.

Structures		Dimensions (mm)	Attenuation (HU)
**Lacrimal gland (lateral-medial and dorsoventral axis)**		4.0–2.0	0–26
**Retrobulbar fat**		-	- 20–100
**Trochlea (base—apex)**		5.0–3.0	-
**Superior oblique muscle**		1.1	35
**Juxtaposed musculature (R—P–O)**		-	60
**Ventral rectus muscle (thickness—caudal)**		0.4–2.4	6 / 14–54
**Medial rectus muscle (near eye—caudal)**		‹ 1.0–2.6	50–55 / 32–66
Optic nerve (NOL and NOR)*–dorsoventral axis		3.1	15–28 / until 40
**Eye (3 external layers)**		0.8–1.2	10–47
**Vitreous chamber (vitreous humor)**		-	0–20
**Aqueous humor**		-	- 2–10
**Eye—LE and RE** **Transverse plane**	Dorsoventral axis	20.2–20.1	-
	Equatorial axis	19.5	-
**Eye—LE and RE** **Sagittal plane**	Rostro-caudal axis	17.3	-
	Dorsoventral axis	19.9	-
**Lens**	Dorsoventral axis	7.9	54–85 (LE)
	Latero-medial axis	7.2	55–101 (RE)

**mm**—millimeters; **HU**—Hounsfield Unit; **LON**—left optic nerve; **RON**—right optic nerve; **R**—rectus superior muscle; **P**—levator palpebrae superioris; **O**—superior oblique muscle; **LE**—left eye; **RE**—right eye.

The ventral rectus muscle is inserted into the eye in ventral portion, attached to the bulbar conjunctiva, and its thickness and attenuation varied according to location. Near the caudal pole of the eye, the muscle separates from the bulbar conjunctiva, presenting a tubular aspect with a greater thickness.

The medial rectus muscle was initially identified from a remnant tissue on the tendon topography of this muscle. The difficulty in identifying the medial and lateral rectus muscles, since its insertion, was due to the eye proximity to the orbit bones, whose distance was less than 1.0 mm. This indicates that the tendon region of the insertion of these muscles also should be very thin.

It is noteworthy the contrast examination although it enhanced the muscle tissue, did not contribute to the individualization and evaluation of the muscles. Fig 1B shows the arrangement of muscles at the apex of the orbit.

#### Optic nerve

Retrobulbar fat surrounding the optic nerve is hyperattenuating, and presents a tubular appearance (Fig 1B). Near the optical channel it fuses to the muscles and forms an optic muscle cone. The dimensions and degree of attenuation are shown in Table 1.

#### Eye

Observed as a single layer of hyperattenuating tissue in relation to its content, with a smooth and regular surface (Fig 1C) and rounded lens (Fig 1D). The dimensions of the eye and its internal structures are shown in Table 1.

#### Orbit

The CT images in the bone tissue window, for analysis of the orbital bones, revealed the orbit limits and their dimensions, observed in Table 2. In transversal plane, the three-dimensional reconstruction image showed the orbit completely encompassed by bone with circular aspect around of the eye (Fig 1E).

**Table 2:** Dimensions and limits of the orbit.

Orbital bones	Dimensions (mm)*
**Maxillary (orbital face)**	0.7
**Ethmoid (rostral—caudal portions)**	1.1–0.4
**Lacrimal**	0.9
**Frontal (orbital face—dorsoventral axis–+ thick)**	0.8–5.7–1.1
**Zygomatic**	3.7
**Sphenoid (greater thickness—caudal portion)**	1.6–0.9
**Palatine**	0.8
Equatorial axis (latero-medial)–LO and RO*	21.2–20.6
Dorsoventral axis (height)–LO and RO*	25.0
**Between the orbits (medially)**	5.3
**Between ethmoid bones**	1.8
Between orbit and eye (D, V, L e M)*	2.8–1.3–0.9–0.7

*mm—millimeters; **LO**—left orbit; **RO**—right orbit; **D**—dorsal; **V**—ventral; **L**—lateral; **M**—medial.

There was variation between the bone densities. Table 3 shows the attenuation degrees of the orbital bones.

**Table 3:** Attenuation degree of the orbital bones.

Orbit bones and regions	Attenuation degree (HU)*
**Frontal**	700–2000 (1358)
**Frontozygomatic Suture**	800
**Zygomatic**	1000–2000
**Maxillary**	400–750
**Lacrimal**	400–900
**Etmomaxilar Suture**	150
**Ethmoid**	250–500
**Sphenoid**	600–1000

* **HU**—Hounsfield Unit.

### Anatomical description of the adnexa and eye

**Fig 2:**
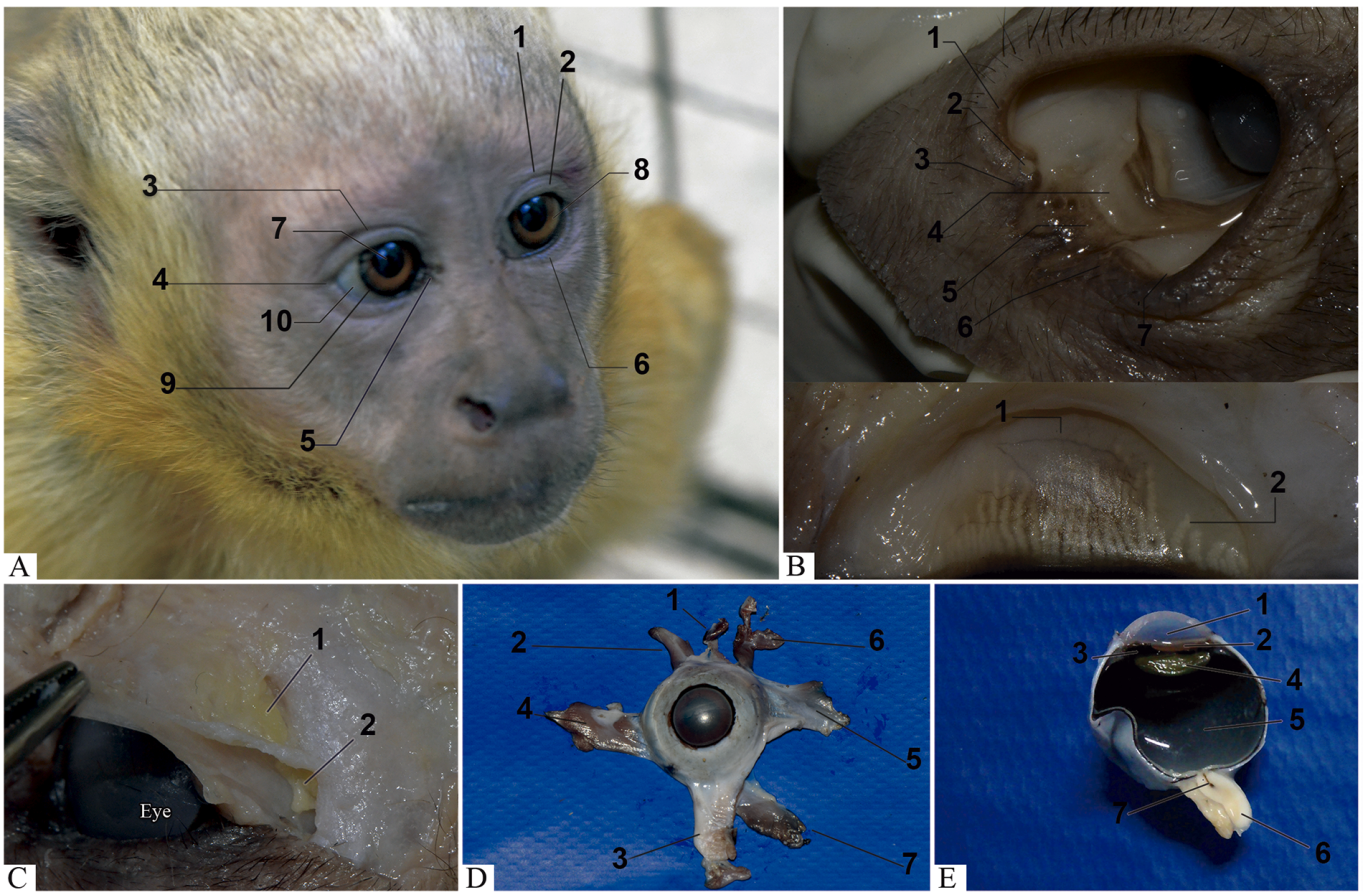
Capuchin monkey. (A) Identification of eyelids (upper eyelid: 1- supratarsal and 2- tarsal parts, 3- upper palpebral groove, 4- lateral commissure of eyelids, 5- medial commissure of eyelids and 6- lower eyelid), arrows indicate each region and external structures of the eye (7- pupil, 8- iris, 9- limbus cornea and 10- sclera). (B) External structures of the eyelids (1- duct of lacrimal glands, 2- lacrimal point, 3- superior lacrimal papilla, 4- semilunar fold of conjunctiva, 5- lacrimal caruncle, 6- inferior lacrimal papilla and 7- anterior limb of eyelid). Posterior aspect of the eyelid (1- conjunctival tunica of the eyelid and 2- tarsal or Meibomian glands). The arrows indicate each region. Evaluation after fixation of formalin structures. (C) Lacrimal gland: 1- orbital and 2- eyelid part. (D) Eye of Sapajus sp. with extrinsic muscles and 1- elevator of upper eyelid inserted. Front view location of the eye and organized muscles in the eye. Note the muscles responsible for the movement of the eye are four rectus muscles: 2- dorsal, 3- ventral, 4- lateral and 5- medial and two oblique muscles: 6- dorsal and 7- ventral. (E) Internal structures of the eye fixed in formalin, sagittal cut. 1- Anterior chamber: note the detail showing the anterior face of the 2- iris. 3- Posterior chamber: observe the 4- lens adhered to the zonular fibers. 5- Vitreous chamber. 6- Optic nerve: observe the 7- central artery of the retina in the center.

The external evaluation of the eyelids and the eye are shown in Fig 2A and 2B. The lacrimal gland was identified in the superolateral region of the orbital cavity. First, after removal of the orbital septum, it was initially observed in the orbital part, and then the eyelid part, presenting a slightly lobulated, yellowish appearance. After fixation in the formalin the dimensions were of 3.56 mm length, 11.35 mm width and 1.62 mm thick (Fig 2C).

After exenteration, retrobulbar fat was observed, especially in the ventral portion of the eye, as well as involving the extraocular and the elevator of upper eyelid muscles, grouped in the posterior pole. The location of extraocular muscles and that of the upper eyelid were identified from their insertion into the eye (Fig 2D). Measurements were made regarding the width, thickness and length of the three animals. In two animals, the distance from the insertion of the muscle in the eye to the limbus was measured. This information is shown in Table 4.

**Table 4:** Dimensions of the extrinsic muscles of the eye and the elevator of eyelid in *Sapajus* sp.

Muscle Capuchin monkey		Dimensions (mm)											
		Length			Width			Thickness			Distance M-L		
		1^a^	2	3	1^a^	2	3	1^a^	2	3	1^a^	2	3
**Dorsal rectus**	**R**	14.20	12.21	12.06	5.58	5.11	5.81	2.16	1.59	1.52	-	9.56	10.57
	**L**	-	11.32	18.67	-	2.74	4.41	-	0.83	1.90	-	8.59	10.49
**Ventral rectus**	**R**	11.50	18.95	8.40	5.19	3.66	3.64	2.69	1.57	1.78	-	8.16	7.92
	**L**	-	12.16	18.65	-	5.88	3.88	-	1.37	2.18	-	8.93	8.66
**Lateral rectus**	**R**	13.28	10.60	7.17	7.70	6.37	6.41	2.19	1.66	0.42	-	11.47	9.34
	**L**	-	8.07	15.44	-	5.07	5.46	-	1.76	1.24	-	11.50	13.96
**Medial rectus**	**R**	11.06	10.90	11.99	7.29	5.63	5.75	3.18	1.60	1.25	-	5.47	7.90
	**L**	-	5.60	15.78	-	4.70	6.80	-	1.45	1.74	-	8.28	9.11
**Dorsal oblique**^b^	**R**	11.09	-	-	4.15	-	-	1.78	-	-	-	-	-
	**L**	-	-	-	-	-	-	-	-	-	-	-	-
**Ventral oblique**	**R**	13.72	14.17	17.32	5.96	6.59	8.39	1.90	1.21	1.23	-	12.74	12.93
	**L**	-	4.40	11.88	-	9.70	6.25	-	1.37	1.08	-	10.19	15.05
**Lev. palp. superioris**	**R**	14.41	10.28	13.11	4.72	6.98	6.26	0.86	0.62	2.43	-	7.24	6.71
	**L**	-	12.45	14.16	-	8.02	3.14	-	0.96	0.62	-	6.74	13.40

**Distance M-L**: distance between the muscle and the limbus; **R**: right; **L**: left; **mm**: millimeters.

^**a**^Capuchin monkey 1 were performed only the measurements of the right eye and was not dimensioned the distance between the muscles and the limbus.

^**b**^**Dorsal oblique**—Capuchin monkeys 2 and 3 were not sized due to the difficult of preserving this structure during dissection.

In the eye "in loco", the sclera is little exposed and the region in which it is visible appears slightly pigmented and brownish. All the rest of this structure has the white color. The sclera is covered by the bulbar conjunctiva. The cornea is transparent and translucent and through it the brownish iris could be visualized without areas of distinct coloration, with a central round pupil. In the corneal periphery, limb and sclera could be observed. In a sagittal section of the eye, the internal structures that compose the anterior, posterior and vitreous chambers are shown in Fig 2E.

### Anatomical description of the orbit

**Fig 3:**
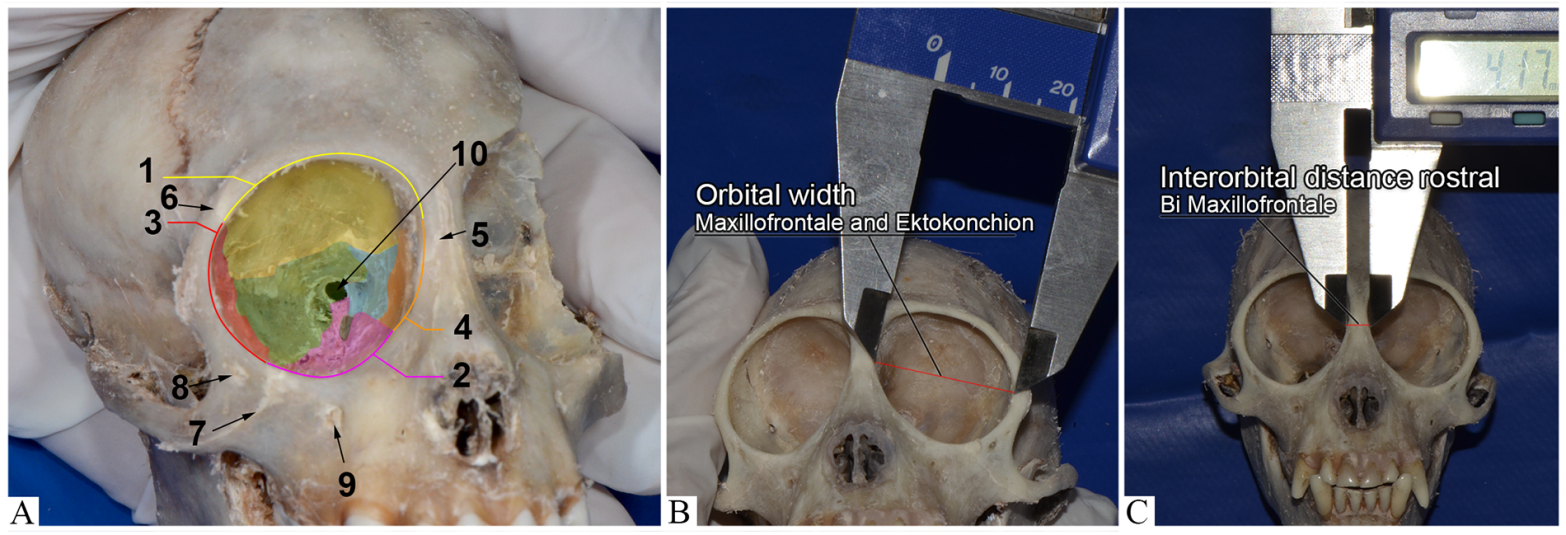
Capuchin monkey macerated head (*Sapajus* sp.). (A) Bones that compose the orbit: frontal (yellow); zygomatic (red); maxillary (pink); lacrimal (orange); ethmoid (blue); sphenoid (green); palatine (gray). Walls of the orbit: 1- dorsal: frontal bone and minor sphenoid wing (yellow); 2- ventral: Maxillary, zygomatic and orbital processes of the palatine bone (pink); 3- lateral: Greater wing of sphenoid bone and zygomatic bone (red) and 4- medial: lacrimal, ethmoid and minor sphenoid wing bones (orange). Orbit limits: supraorbital border, zygomatic bone, frontal bone, maxillary bone, infraorbital border, anterior lacrimal crest. Sutures: 5-frontomaxillary, 6- frontozygomatic and 7- zygomaticomaxillary. Foraminas: 8- zygomaticofacial infraorbital and 9- maxillary infraorbital. The supraorbital foramina was not observed. Note the 10- optical channel. (B) Orbit width—distance from maxillofrontale point (mf) to ektokonchion point (ek). (C) Linear magnitude of the face. Orbit measurement—Rostral interorbital width or Bi Maxillofrontale (red line).

In all of the studied animals, an orbital pyramid or cone shape with the apex facing the posterior region was observed. The anterior region presented with rounded and thick edges, forming a complete orbit, consisting of seven individual bones that form four walls (Fig 3A).

In a frontal view, we observe the orbital adit, with the dorsal, ventral lateral and medial borders, orbital sutures and foramina seen in Fig 3A. Information related to the distances between these foramina are described in Table 5.

**Table 5:** Distances of infraorbital foramina in *Sapajus* sp.

Infraorbitarios foramina (Distances)	Capuchin monkey		
	1	2	3
**Distance between (zygomaticofacial-maxilar)**	8.09 mm (RE)	7.32 mm (RE)	9.31 mm (RE)
	8.09 mm (LE)	7.36 mm (LE)	9.31 mm (LE)
**Distance between the edge of the maxilla and maxillary foramina**	2.96 mm (RE)	2.84 mm (RE)	3.11 mm (RE)
	2.96 mm (LE)	3.0 mm (LE)	3.28 mm (LE)
**Distance between the zygomaticofacial and the zygomatic process of the maxilla**	3.08 mm (RE)	2.98 mm (RE)	4.95 mm (RE)
	3.08 mm (LE)	2.86 mm (LE)	4.81 mm (LE)

Dimensions of the orbit of the three animals are described in Tables 6 and 7 shown from the representative images (Fig 3B and 3C).

**Table 6:** Dimensions of the orbit in *Sapajus* sp. Linear magnitude of the face—Orbital region.

Parameters	Capuchin monkey	Right orbit	Left orbit
**Orbit height**	**1**	20.05 mm	20.22 mm
	**2**	20.40 mm	20.74 mm
	**3**	22.35 mm	22.35 mm
**Orbit width Maxillofrontale**^1^ **and Ektokonchion**^2^	**1**	18.82 mm	18.82 mm
	**2**	20.95 mm	20.95 mm
	**3**	22.00 mm	21.96 mm
**Orbit index (%)**	**1**	93.87%	93.08%
	**2**	102.70%	101.01%
	**3**	98.43%	98.25%
**Depth of orbit**	**1**	23.09 mm	23.07 mm
	**2**	27.58 mm	27.57 mm
	**3**	32.73 mm	29.97 mm
**Orbit area (mm**^**2**^**)**	**1**	296.20 mm^2^	298.71 mm^2^
	**2**	335.33 mm^2^	340.92 mm^2^
	**3**	385.81 mm^2^	385.11 mm^2^

^1^**Maxillofrontale**—Point, in the orbital border, where it is cut by the frontomaxillary suture.

^2^**Ektokonchion**—Point located at the outer edge of the orbit and as far from the maxillofrontal point as possible.

**Table 7:** Dimensions of the orbit in three *Sapajus* sp. Linear magnitude of the face—Interorbital region.

Parameters	Capuchin monkey		
	1	2	3
**Bi-orbital distance Bi Ektokonchion**^1^	46.54 mm	41.37 mm	46.99 mm
**Rostral interorbital distance** **[Bi Maxillofrontale**^2^**]**	4.79 mm	4.17 mm	5.21 mm
**Medial interorbital distance** **[Bi Lacrimale**^3^**]**	5.53 mm	4.34 mm	6.49 mm
**Caudal interorbital distance** **[Bi Dakryon**^4^**]**	4.82 mm	5.09 mm	6.22 mm

^**1**^**Ektokonchion**—Point located at the outer edge of the orbit and as far from the maxillofrontal point as possible.

^**2**^**Maxillofrontale**—Point, in the orbital border, where it is cut by the frontomaxillary suture.

^**3**^**Lacrimale**—Point of encounter of the posterior lacrimal crest with the frontal.

^**4**^**Dakryon**—Point located at the vertex of the angle formed by the suture frontolacrimale the lacrimomaxilar suture.

### Histological evaluation of the adnexa and eye

#### Eyelid

**Fig 4:**
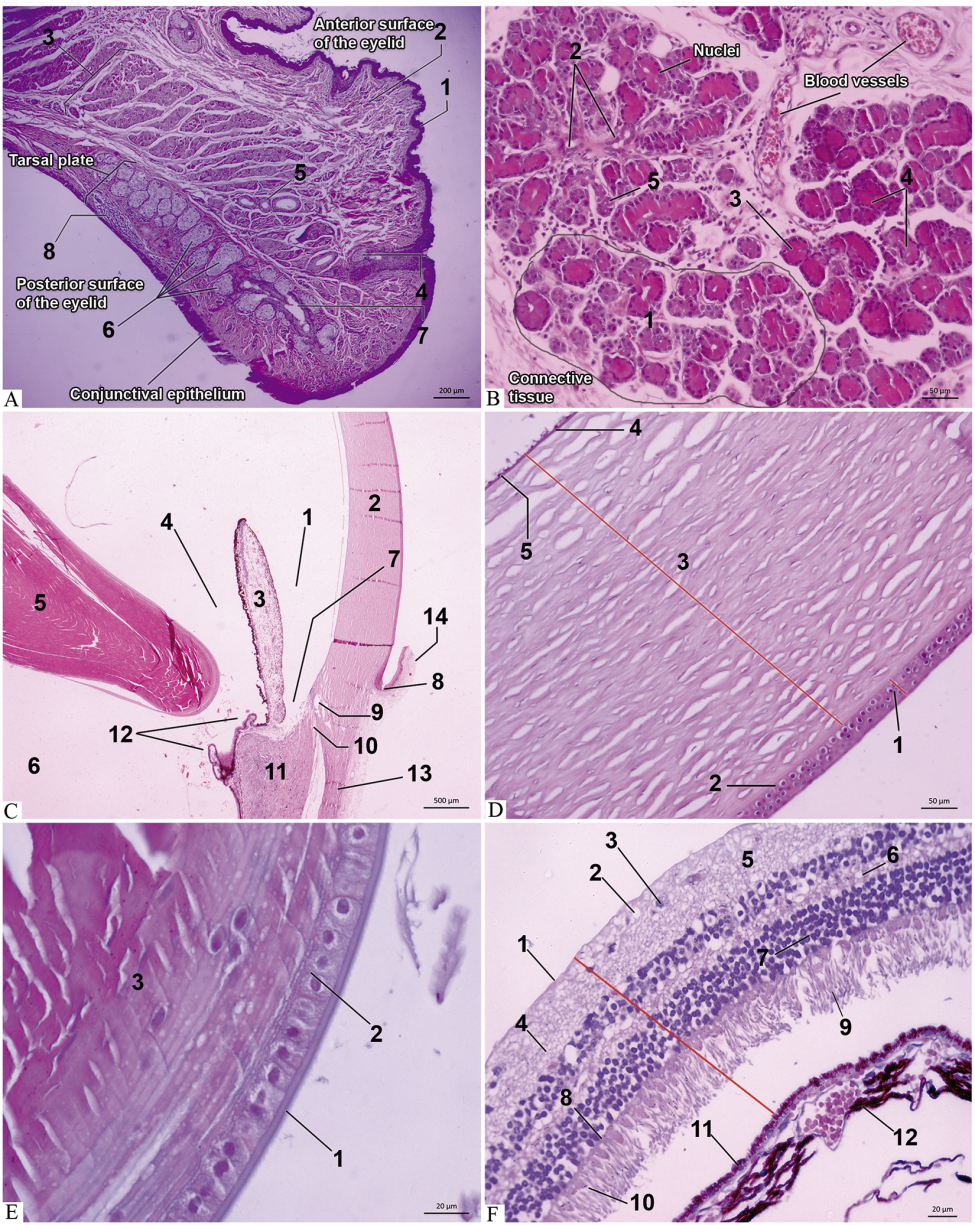
Photomicrography of the eye and adnexa of the capuchin monkey. (A) Eyelids: Observe all the structures that comprise it: 1- epidermis, 2- dermis, 3- skeletal striated muscle fibers of the palpebral part of the orbicularis oculi and pilous follicles (note the 4- Zeis glands at the base of the follicle), 5- Moll glands, 6- tarsal glands, 7- excretory duct of the gland Tarsal and 8- lymphoid follicle. HE. Obj. 5x. (B) Lacrimal gland: glandular tubuloalveolar parenchyma forming 1- lobules and lobes composed of 2- ducts and numerous 3- serous acini, responsible for the 4- secretory portion. Note the 5- amphiphilic granules. HE. Obj. 20x. (C) Eye: 1- anterior chamber (space between the posterior surface of the 2- cornea and the anterior face of the 3- iris); 4- posterior chamber (located between the posterior face of the iris and 5- lens) e a 6- vitreous chamber (space after the lens). Observe the 7- iridocorneal angle (located between the cornea and the iris), 8- limbus (9- Schlemm's canal, 10- trabecular meshwork adjacent to the canal), 11- ciliary body, 12- ciliary processes, 13- scleral and 14- bulbar conjunctiva. HE. Obj. 2,5x. (D) Cornea: layers– 1- anterior epithelium, 2- Bowman's membrane, 3- stroma, 4- Descemet's membrane and 5- endothelium. HE. Obj. 20x. (E) Lens: layers– 1- capsule of the lens, 2- subcapsular epithelium and 3- lamellar fibers. HE. Obj. 63x. (F) Retina: 1- inner limiting membrane, 2- optic never fibers layer, 3- ganglion cell layer, 4- inner plexiform layer, 5- Inner nuclear layer, 6- outer plexiform layer, 7- external nuclear layer, 8- outer limiting membrane, 9- rods (basophylic and elongated), and 10- cones (basophilic and oval). Note the 11- pigment epithelium of the retina adhered to Bruch's membrane of the 12- choroid. HE. Obj. 40x.

Zeis’ glands were identified at the base of the pilous follicles. The apocrine sweat glands called Moll glands or ciliary glands were located near these follicles. Other structures that compose the eyelids are identified in Fig 4A.

#### Lacrimal gland

Parenchyma formed by tubuloalveolar serous glands, separated into lobes and lobules that connective tissue elements (Fig 4B).

#### Extraocular muscles

The cross-sectional histological sections reveal striated skeletal musculature, individualized by three layers of dense connective tissue, the epimysium, perimysium and endomysium. Muscle fibers are divided in two "red" and "white" types. The longitudinal histological sections showed the transverse striations and the bands A and I.

#### Eye

Histologically the anterior, posterior and vitreous chambers can be seen in Fig 4C. The cornea consists five layers: anterior epithelium, Bowman's membrane, stroma, Descemet's membrane and endothelium (Fig 4D). The iris has a free border and is directly attached to the ciliary body, where the ciliary muscle was identified, and the pars plicata, ciliary processes was sometimes undeveloped. The lens consists of a predominantly eosinophilic structure, divided in three layers (Fig 4E).

The sclera is formed by bundles of collagens organized in parallel and interlaced. The choroid is attached anteriorly to the ciliary body and posterior to the margin of the optic nerve. It is formed by the supracoroid space, larger vessels, smaller vessels, Bruch's membrane and retinal pigment epithelium. The histological sections of the retina showed that it is formed by ten layers (Fig 4F).

In the sagittal section of the optic nerve, the continuation of the retina, choroid and its structures, such as the excavation papilla of the optic nerve is observed.

## Discussion

The anatomical study of the adnexa, eye and orbit in *Sapajus* sp. by CT scan in the live capuchin monkey and in the cadaver revealed no structural differences. It is emphasized that the use of contrast in the live animal that would aim to highlight the structures visualization in soft tissue window did not influence the reading and interpretation of results in this study. However, a study carried out on some reptile species with radiography and computed tomography considered that the use of contrast allowed a better visualization of structures in the soft tissue window [17].

The observed retrobulbar fat enveloping the extraocular muscles made it possible to identify them, since fat presented hypoattenuating on computed tomography, creating a contrast with the structure that is adhered to and facilitating the identification, as already reported by other authors in humans and reptiles [17–21]. The sclera, choroid and retina structures of the eye were observed as a monolayer. However other structures such as the lens, aqueous humor and vitreous humor were identified as described in other studies [17,20].

The macroscopic evaluation of the four rectus extraocular muscles: dorsal, ventral, lateral and medial and two oblique: dorsal and ventral, of capuchin monkey, are in number and location similar to those of the human species [22–24] and PNH [25]. However, there were differences in the width dimensions of the capuchin monkey muscles in relation to the subspecies of gorilla and the human species [7,26]. Histologically, they present epimysium, perimysium, endomysium, striated muscle fibers with peripheral nuclei, collagen fibers interspersed with muscle fibers, fibroblasts and bundles of nerve fibers, structures that characterize musculature as skeletal striatum [27–29].

The lacrimal gland presented similar localization to that observed in humans [22,24] and other species, such as canines and felines [30] and, unlike the guinea pig that does not have the supraorbital but infraorbital lacrimal gland [31]. Its identification in the anatomical part was facilitated after the CT scan that demonstrated its location and size [20]. Histology has similar characteristics as those described in the literature, such as serous glands of the tubuloalveolar type, the formation of lobes that are composed of numerous serous acini, with round nuclei located at the base of these cells and sometimes amphiphilic secretory granules [27–29].

Similarity of anatomical evaluation of the eyelids in this study was observed with the structures present in the human species, PHN and domestic species. Except for the presence of a vestige of third eyelid or nictitating membrane, common in domestic species [32] and variable in primates. The nictitating membrane in primates may cover the entire eye, such as animals of the genus *Arctocebus* and *Perodicticus*, but may also be absent as with *Pan* and *Homo* animals [25,33,34].

Anatomopathological study of ocular structures of the capuchin monkey revealed many similarities and subtle differences with the humans. There was little exposure of the sclera and it was slightly brownish with greater intensity in the region near the corneal limbus. Kobayashi and Kohshima [35] identified this variation in sclera staining and classified it as an adaptive function. There are reports that pigmentation of the sclera may help these animals escape the attacks of other individuals [36].

The relationship between sclera color, iris color and the color of the face around the eye in PHN and the humans were classified in four types according to the difference in color or contrast between sclera and iris or the face. In this classification, the capuchin monkey is inserted in type I (face ~ sclera ~ iris), the dark color of the sclera is similar to face and iris color and the human in type IV (face> sclera <iris), that the color of the sclera is paler than the color of the face and iris [35]. No differences were observed in the internal structures of the eye, iris, ciliary body, *ora serrata*, ciliary processes, lens, optic nerve when compared with the human eye [37–39].

The bony structures that compose the orbit (frontal, zygomatic, sphenoid, maxillary, palatine, ethmoid and lacrimal) of *Sapajus* sp. are similar to the structures of the other domestic and human species [22,23], [40–42]. However, discrete differences were observed as absence of the supraorbital foramen, located near the supraorbital margin and absence of the frontal notch in the antero-medial region of the supraorbital margin.

The foramen and fissures present in the cranial cavity are access routes of blood vessels and nerves from the brain to the eye [13]. These perforations may be fused in some species and this fusion results in changes in the course of a particular vessel or nerve in the various species [43]. However, the absence or fusion of the foramen does not compromise the neurovascular supply of the region to be supplied, since there is an alternative route. Other authors report asymmetry in the localization of the foramina or even one-sidedness of them in the human species [44–47].

In this anatomical study, it was observed that the orbit in the capuchin monkey has a more rounded shape in the base or ventral wall, when compared with man, bovine, goat and swine [48–51]. The size of the opening of the orbit in primates is related to the habits or activity of these animals, although other factors such as the size and orientation of the eye and the size of the orbit can also influence [52]. Day-time animals have smaller orbital apertures than nocturnal primates. This is due to the fact that nocturnal primates have larger eyes, so there is a need to have a larger orbit for adequate accommodation [53–56]. These animals have greater visual acuity because they present a large cornea and lens [52].

A marked variation was observed between orbital parameters of capuchin monkey in relation to the parameters in humans [22,24]. This marked variation of the parameters is probably related to the height of the individuals, considering the size of the skulls and consequently the orbit of *Sapajus* sp. be smaller when compared to man.

The microscopic findings in this study describe the cornea composed of five layers: anterior epithelium, Bowman's membrane, stroma, Descemet's membrane and corneal endothelium, something observed in most species. In the study of anatomy and comparative ocular histology performed by Knapp et al. [7] with the gorilla and the human species he also describes the same structures in these species. However, Dua et al. [57] identified a sixth layer in the cornea in humans which they called pre-Descemet or *Dua's Layer*, according to these authors this layer is more resistant. From this discovery they have suggested a redefinition of human cornea structures.

The corneal epithelium in the capuchin monkey is composed of three to four layers, a finding similar to the western lowland gorilla [7]. However, in man the corneal epithelium has five to six layers of cells [58].

The difference observed in the corneal epithelium thickness of the capuchin monkey, which is thinner in relation to humans, does not cause clinical interference, according to studies of reference values for diagnostic ophthalmic tests in capuchin monkey, where the values of the central corneal thickness for the adult capuchin monkey was similar to that found for humans using ultrasonic pachymetry (0.467 ± 0.040 mm) [59,60].

In this study, the histological analysis of the vascular layer, iris, ciliary body and choroid of *Sapajus* sp. revealed hyperpigmentation of these structures in relation to other species including man [7]. In PHN the choroid is thinner and abundant in large melanocytes, which gives it a darker color [61].

Histologically, the capuchin monkey retina presents all layers with the same typical pattern observed in mammals [9,30,62,63]. The neurosensory layer as well as other species varies in thickness along the vitreous chamber, thicker near the optic nerve and thinner in the ciliary region (*ora serrata*) [30]. The outermost layer, the pigment epithelium appears strongly adhered to the Bruch's membrane on the choroid, this layer is responsible for the blood supply of the retina coming from the choroid [30]. The vascular pattern of the retina has the holangiotic type, with the distribution of the main retinal vessels from the optic papilla, and retinal asymmetry has also been described by other authors [62,63].

## Conclusions

The computerized tomography technique can be used as a diagnostic tool in the evaluation of the adnexa, eye and orbit, both in the live animal and in the cadaver without loss of visibility of the structures. In addition, to facilitating the anatomical identification of certain structures, such as the lacrimal gland. Anatomically, discrete differences were observed in relation to the human being, characterized by little exposure of the sclera, slightly pigmented sclera with marked pigmentation at the sclerocorneal junction, absence of the supraorbital foramen, frontal notch and more rounded orbit.

Histological differences with the human species were minimal and related to the cell layer number of the corneal epithelium and hyperpigmentation of the vascular layer. The *Sapajus* sp. proved to be an important experimental model for research in the field of ophthalmology due to the high similarity of theses anatomical and histological structures with the human species.

## Supporting information

S1 File CEUA—Ethics Commission for the use of Experimental Animals. DOI: https://doi.org/10.6084/m9.figshare.5538916.v1.
(PDF)

S2 File SISBIO—Authorization and Information System on Biodiversity. DOI: https://doi.org/10.6084/m9.figshare.5538934.v1.
(PDF)
