# Supplementary material for: Morphological study of the eye and adnexa in capuchin monkeys (Sapajus sp.)
Source: PLoS One. 2017 Dec 5;12(12):e0186569. doi: 10.1371/journal.pone.0186569 (PMC5716594; doi:10.1371/journal.pone.0186569)
Supplement: S1 File — DOI: https://doi.org/10.6084/m9.figshare.5538916.v1. (PDF) [file pone.0186569.s001.pdf]

Universidade Federal da Bahia
Escola de Medicina Veterinária e Zootecnia
Comissão de Ética no Uso de Animais
Av. Ademar de Barros, 500 – Ondina-40170-110 Salvador-BA
Fone: (071) 3283-6704/6708/ - Fax: 3283-6718
E-mail: escmev@ufba.br

CERTIFICADO

Cetificamos que o projeto de pesquisa intitulado **"Estudo anatomohistológico do bulbo ocular e anexos de Sapujos sp",** protocolo nº 42/2015 sob a responsabilidade da pesquisadora **Alessandra Estrela da Silva Lima** e que envolve a produção, manutenção e/ou utilização de animais pertencentes ao filo Chordata, subfilo Verbrata (exceto o homem), para fins de pesquisa cientifica, encontra-se de acordo com os preceitos da Lei nº 11.794, de 8 de outubro de 2008, do Decreto nº 6.899, de 15 de julho de 2009, e com as normas editadas pelo Conselho Nacional de Controle da Experimentação Animal (CONCEA), e foi aprovado pela Comissão de Ética no Uso de Animais (CEUA), da Escola de Medicina Veterinária e Zootecnia da Universidade Federal da Bahia, em reunião de 23 de outubro de 2015.

Vigência do projeto	20/10/2015 a 20/07/2016
Especie/linhagem	Primata não humano, linhagem híbrido
Nº de aninais	10 (dez)
Peso/Idade	1,5 a 3,0 Kg / idade desconhecida
Sexo	Indeterminado
Origem	Centro de triagem de Animais Silvestres (CETAS).

Salvador, 27 de outubro de 2015.

Lúcio Leopoldo de Aragão da Silva
Coordenador da CEUA/MEVZ/UFBA
