# Supplementary material for: Morphological study of the eye and adnexa in capuchin monkeys (Sapajus sp.)
Source: PLoS One. 2017 Dec 5;12(12):e0186569. doi: 10.1371/journal.pone.0186569 (PMC5716594; doi:10.1371/journal.pone.0186569)
Supplement: S2 File — DOI: https://doi.org/10.6084/m9.figshare.5538934.v1. (PDF) [file pone.0186569.s002.pdf]

Ministério do Meio Ambiente - MMA
Instituto Chico Mendes de Conservação da Biodiversidade - ICMBio
Sistema de Autorização e Informação em Biodiversidade - SISBIO

Autorização para atividades com finalidade científica

Número: 51786-1	Data da Emissão: 22/08/2016 09:13	Data para Revalidação*: 21/09/2017

* De acordo com o art. 28 da IN 03/2014, esta autorização tem prazo de validade equivalente ao previsto no cronograma de atividades do projeto, mas deverá ser revalidada anualmente mediante a apresentação do relatório de atividades a ser enviado por meio do Sisbio no prazo de até 30 dias a contar da data do aniversário de sua emissão.

Dados do titular

Nome: Alessandra Estrela da Silva Lima	CPF: 899.826.575-34
Título do Projeto: Estudo descritivo anatômico e histológico de olho e anexos em macaco-prego (Sapajus sp.).	
Nome da Instituição : UNIVERSIDADE FEDERAL DA BAHIA	CNPJ: 15.180.714/0001-04

SISBIO

Cronograma de atividades

#	Descrição da atividade	Início (mês/ano)	Fim (mês/ano)
1	Colheita e preparação das amostras	12/2016	12/2017
2	Documentação fotográfica das estruturas	02/2017	11/2017
3	Avaliação histológica	02/2017	11/2017
4	Descrição anatômica	02/2017	11/2017
5	Processamento histológico	02/2017	11/2017
6	Dissecação anatômica	02/2017	11/2017
7	Descrição histológica	02/2017	12/2017

Observações e ressalvas

1	As atividades de campo exercidas por pessoa natural ou jurídica estrangeira, em todo o território nacional, que impliquem o deslocamento de recursos humanos e materiais, tendo por objeto coletar dados, materiais, espécimes biológicos e minerais, peças integrantes da cultura nativa e cultura popular, presente e passada, obtidos por meio de recursos e técnicas que se destinem ao estudo, à difusão ou à pesquisa, estão sujeitas a autorização do Ministério de Ciência e Tecnologia.
2	Esta autorização NÃO exime o pesquisador titular e os membros de sua equipe da necessidade de obter as anuências previstas em outros instrumentos legais, bem como do consentimento do responsável pela área, pública ou privada, onde será realizada a atividade, inclusive do órgão gestor de terra indígena (FUNAI), da unidade de conservação estadual, distrital ou municipal, ou do proprietário, arrendatário, posseiro ou morador de área dentro dos limites de unidade de conservação federal cujo processo de regularização fundiária encontra-se em curso.
3	Este documento somente poderá ser utilizado para os fins previstos na Instrução Normativa ICMBio n° 03/2014 ou na Instrução Normativa ICMBio n° 10/2010, no que especifica esta Autorização, não podendo ser utilizado para fins comerciais, industriais ou esportivos. O material biológico coletado deverá ser utilizado para atividades científicas ou didáticas no âmbito do ensino superior.
4	A autorização para envio ao exterior de material biológico não consignado deverá ser requerida por meio do endereço eletrônico www.ibama.gov.br (Serviços on-line - Licença para importação ou exportação de flora e fauna - CITES e não CITES).
5	O titular de licença ou autorização e os membros da sua equipe deverão optar por métodos de coleta e instrumentos de captura direcionados, sempre que possível, ao grupo taxonômico de interesse, evitando a morte ou dano significativo a outros grupos; e empregar esforço de coleta ou captura que não comprometa a viabilidade de populações do grupo taxonômico de interesse em condição in situ.
6	O titular de autorização ou de licença permanente, assim como os membros de sua equipe, quando da violação da legislação vigente, ou quando da inadequação, omissão ou falsa descrição de informações relevantes que subsidiaram a expedição do ato, poderá, mediante decisão motivada, ter a autorização ou licença suspensa ou revogada pelo ICMBio, nos termos da legislação brasileira em vigor.
7	Este documento não dispensa o cumprimento da legislação que dispõe sobre acesso a componente do patrimônio genético existente no território nacional, na plataforma continental e na zona econômica exclusiva, ou ao conhecimento tradicional associado ao patrimônio genético, para fins de pesquisa científica, bioprospecção e desenvolvimento tecnológico. Veja maiores informações em www.mma.gov.br/cgen.
8	Em caso de pesquisa em UNIDADE DE CONSERVAÇÃO, o pesquisador titular desta autorização deverá contactar a administração da unidade a fim de CONFIRMAR AS DATAS das expedições, as condições para realização das coletas e de uso da infra-estrutura da unidade.

Equipe

#	Nome	Função	CPF	Doc. Identidade	Nacionalidade
1	Danielle Nascimento Silva	Pesquisadora	011.075.365-88	0880779934 SSP-BA	Brasileira

Locais onde as atividades de campo serão executadas

#	Município	UF	Descrição do local	Tipo
1	SALVADOR	BA	Centro de Triagem de Animais Silvestres (CETAS/SALVADOR)	Fora de UC Federal

Este documento (Autorização para atividades com finalidade científica) foi expedido com base na Instrução Normativa nº 03/2014. Através do código de autenticação abaixo, qualquer cidadão poderá verificar a autenticidade ou regularidade deste documento, por meio da página do Sisbio/ICMBio na Internet (www.icmbio.gov.br/sisbio).

SISBIO

Código de autenticação: 46398876

Autorização para atividades com finalidade científica

Número: 51786-1	Data da Emissão: 22/08/2016 09:13	Data para Revalidação*: 21/09/2017

* De acordo com o art. 28 da IN 03/2014, esta autorização tem prazo de validade equivalente ao previsto no cronograma de atividades do projeto, mas deverá ser revalidada anualmente mediante a apresentação do relatório de atividades a ser enviado por meio do Sisbio no prazo de até 30 dias a contar da data do aniversário de sua emissão.

Dados do titular

Nome: Alessandra Estrela da Silva Lima	CPF: 899.826.575-34
Título do Projeto: Estudo descritivo anatômico e histológico de olho e anexos em macaco-prego (Sapajus sp.).	
Nome da Instituição : UNIVERSIDADE FEDERAL DA BAHIA	CNPJ: 15.180.714/0001-04

SISBIO

Atividades X Táxons

#	Atividade	Táxons
1	Coleta/transporte de amostras biológicas ex situ	Primates, Sapajus

Material e métodos

1	Amostras biológicas (Primatas)	Fragmento de tecido/órgão, Animal encontrado morto ou partes (carcaça)/osso/pele
2	Método de captura/coleta (Primatas)	Outros métodos de captura/coleta(animais que foram a obito em CETAS)
3	Método de marcação (Primatas)	Outros métodos de marcação

Destino do material biológico coletado

#	Nome local destino	Tipo Destino
1	UNIVERSIDADE FEDERAL DA BAHIA	

Este documento (Autorização para atividades com finalidade científica) foi expedido com base na Instrução Normativa nº 03/2014. Através do código de autenticação abaixo, qualquer cidadão poderá verificar a autenticidade ou regularidade deste documento, por meio da página do Sisbio/ICMBio na Internet (www.icmbio.gov.br/sisbio).

SISBIO

Código de autenticação: 46398876

Autorização para atividades com finalidade científica

Número: 51786-1	Data da Emissão: 22/08/2016 09:13	Data para Revalidação*: 21/09/2017

* De acordo com o art. 28 da IN 03/2014, esta autorização tem prazo de validade equivalente ao previsto no cronograma de atividades do projeto, mas deverá ser revalidada anualmente mediante a apresentação do relatório de atividades a ser enviado por meio do Sisbio no prazo de até 30 dias a contar da data do aniversário de sua emissão.

Dados do titular

Nome: Alessandra Estrela da Silva Lima	CPF: 899.826.575-34
Título do Projeto: Estudo descritivo anatômico e histológico de olho e anexos em macaco-prego (Sapajus sp.).	
Nome da Instituição : UNIVERSIDADE FEDERAL DA BAHIA	CNPJ: 15.180.714/0001-04

SISBIO

Registro de coleta imprevista de material biológico

De acordo com a Instrução Normativa nº 03/2014, a coleta imprevista de material biológico ou de substrato não contemplado na autorização ou na licença permanente deverá ser anotada na mesma, em campo específico, por ocasião da coleta, devendo esta coleta imprevista ser comunicada por meio do relatório de atividades. O transporte do material biológico ou do substrato deverá ser acompanhado da autorização ou da licença permanente com a devida anotação. O material biológico coletado de forma imprevista, deverá ser destinado à instituição científica e, depositado, preferencialmente, em coleção biológica científica registrada no Cadastro Nacional de Coleções Biológicas (CCBIO).

Táxon*	Qtde.	Tipo de amostra	Qtde.	Data

* Identificar o espécime no nível taxonômico possível.

Este documento (Autorização para atividades com finalidade científica) foi expedido com base na Instrução Normativa nº 03/2014. Através do código de autenticação abaixo, qualquer cidadão poderá verificar a autenticidade ou regularidade deste documento, por meio da página do Sisbio/ICMBio na Internet (www.icmbio.gov.br/sisbio).

SISBIO

Código de autenticação: 46398876
